# Hypogonadotropic Hypogonadism due to Novel FGFR1 Mutations

**DOI:** 10.4274/jcrpe.3908

**Published:** 2017-06-01

**Authors:** Gamze Akkuş, Leman Damla Kotan, Erdem Durmaz, Eda Mengen, İhsan Turan, Ayça Ulubay, Fatih Gürbüz, Bilgin Yüksel, Tamer Tetiker, A. Kemal Topaloğlu

**Affiliations:** 1 Çukurova University Faculty of Medicine, Division of Endocrinology, Adana, Turkey; 2 Çukurova University Faculty of Medicine, Division of Pediatric Endocrinology, Adana, Turkey; 3 İzmir University Faculty of Medicine, Division of Pediatric Endocrinology, İzmir, Turkey; 4 Çukurova University Faculty of Medicine, Department of Forensic Medicine, Adana, Turkey

**Keywords:** Hypogonadotropic hypogonadism, FGFR1 mutations, Kallmann syndrome, reduced penetrance

## Abstract

**Objective:**

The underlying genetic etiology of hypogonadotropic hypogonadism (HH) is heterogeneous. Fibroblast growth factor signaling is pivotal in the ontogeny of gonadotropin-releasing hormone neurons. Loss-of-function mutations in FGFR1 gene cause variable HH phenotypes encompassing pubertal delay to idiopathic HH (IHH) or Kallmann syndrome (KS). As FGFR1 mutations are common, recognizing mutations and associated phenotypes may enhance clinical management.

**Methods:**

Using a candidate gene approach, we screened 52 IHH/KS patients.

**Results:**

We identified three novel (IVS3-1G>C and p.W2X, p.R209C) FGFR1 gene mutations. Despite predictive null protein function, patients from the novel mutation families had normosmic IHH without non-reproductive phenotype.

**Conclusion:**

These findings further emphasize the great variability of FGFR1 mutation phenotypes in IHH/KS.

## What is already known on this topic?

Hypogonadotropic hypogonadism is a well-known rare disorder of very variable genetic etiology.

## What this study adds?

This study has shown that FGFR1 mutations cause phenotypic variations.

## INTRODUCTION

Idiopathic hypogonadotropic hypogonadism (IHH) is a rare clinical disorder characterized by delayed or absent pubertal development (1). IHH has an incidence of 1-10 cases per 100,000 births and it is more common in males (2). If a patient with IHH has an impaired sense of smell, then the condition is called Kallmann syndrome (KS). To date, at least 17 genes have been associated with KS and these include *KAL1, FGFR1, PROK2, PROKR2, FGF8, HS6ST1, CHD7, WDR11, SEMA3A, FGF17, IL17RD, DUSP6, SPRY4, FLRT3, NELF, FEZF1,* and *CCDC141* (3).

KS is most commonly caused by mutations in anosmin 1 encoded by *KAL1* (2,3,4,5). *FGFR1* encodes a tyrosine kinase receptor that mediates fibroblast growth factor signaling (6). Presence of various congenital anomalies which are not associated with the reproductive system such as defects in kidney and tooth differentiation, ear and palate morphogenesis and development of interhemispheric or cortico-spinal axonal tracts encountered in a proportion of KS patients due to FGFR1 mutations indicates that the fibroblast growth factor signaling plays important roles in many other developmental processes. The precise roles played by KS genes in these processes are not known (6). *FGFR1* has been shown to be a key factor for angiogenesis, embryogenic development, and wound healing (7,8). FGFR1 knock-out mice do not have telencephalon and have an altered gonadotropin-releasing hormone (GnRH) migration (9). *FGFR1* mutations cause KS/IHH with or without defects in the reproductive system (10).

In this study, aiming to contribute to the genotypic and phenotypic correlation in IHH/KS cases, we report familial cases due to novel FGFR1 mutations.

## METHODS

We screened 52 IHH/KS patients (36 male, 16 female) in our cohort. The majority of patients (n=38) were normosmic IHH (nIHH) and the remaining 12 were cases of anosmic or hyposmic IHH (KS). Diagnosis of IHH/KS was based on delayed or absent spontaneous puberty by age 13 in girls (Tanner breast stage 1) and by age 14 in boys (testicular volume <4 mL). The patients had bone ages of 11.5 years or greater, with concentrations of serum testosterone and estradiol at hypogonadal levels [<20 ng/dL (714 pmol/L) and <1.9 ng/dL (73 pmol/L), respectively] in the setting of inappropriately normal or low serum gonadotropins. Serum levels for thyroid-stimulating hormone (TSH) with free thyroxin (fT_4_), prolactin, insulin-like growth factor-1, adrenocorticotropic hormone, and cortisol were within normal limits. Exclusion criteria included chronic systemic diseases (impaired renal function, thalassemia, poorly controlled diabetes mellitus), eating disorders (bulimia or anorexia nervosa), or structural anomalies on hypothalamo-pituitary imaging. Sense of smell of the probands was tested while subjecting them to a battery of 10 culturally appropriate odors. This study was approved by the Ethics Committee of Çukurova University Faculty of Medicine. Written informed consents were obtained from all subjects.

### Laboratory Methods

Serum concentrations of luteinizing hormone (LH), follicle-stimulating hormone (FSH), total testosterone, estradiol, prolactin, fT_4_, and TSH were determined by immunofluorometric assays. A GnRH stimulation test (2.5 µ/kg, maximum 100 µ, IV) was performed in all probands. Serum LH and FSH levels were measured at 0, 15, 30, 45, and 60 min after GnRH injection.

For molecular genetic studies, genomic DNAs were isolated from white blood cells. The coding and neighboring intronic regions of the known or strong candidate genes for nIHH (*GNRHR, GNRH1, TACR3, TAC3, FGFR1, KISS1R, and KISS1*) or KS (*KAL1, FGFR1, PROK2, PROKR2*) were amplified by polymerase chain reaction (PCR). PCR products were purified and directly sequenced using the BigDye terminator cycle sequencing ready reaction kit (PE Applied Biosystems, Foster City, Calif., USA) in an ABI PRISM 3130 automatic sequencer.

Whole Exome Sequencing was performed at Yale Center for Genome Analysis using NimbleGen 2.1M human exome array (Roche NimbleGen, Inc.) according to the manufacturer’s protocol with certain modifications, as described previously (11). Sequencing of the library was performed on HiSeq2000. The Illumina pipeline version 1.8 was used for image analysis and base calling.

## CASE REPORTS

### Family 1

The proband (II-4) was a 17-year-old female patient who was referred for lack of breast development and primary amenorrhea. Her height and weight were 161 cm (25-50^th^ percentile) and 43 kg (<3^th^ percentile), respectively. She had a normal sense of smell. Her breast development was at Tanner stage 2 at the right and stage 1 at the left breast. Her axillary hair and pubic hair were at stages 3 and 2, respectively.

Her estradiol and gonadotropins were at prepubertal levels. A GnRH stimulation test revealed maximal LH and FSH concentrations of 7.0 and 7.7 mIU/mL, respectively. Chromosome analysis showed a 46,XX karyotype. Her pelvic ultrasonography and cranial magnetic resonance imaging (MRI) results were normal.

One of her sisters (II-2), a 22-year-old female who had complaints of absent breast enlargement and primary amenorrhea and who had been given estrogen treatment in another clinic was also diagnosed as a case of IHH. This patient’s height and weight were 165 cm (50-75^th^ percentile) and 55 kg (25-50^th^ percentile), respectively. Her sense of smell was normal. Her pubic and axillary hair were both at stage 3, while her breast Tanner stage was 4. Her karyotype was 46,XX. Her serum plasma estradiol, LH, and FSH levels were prepubertal (Table 1). Their parents were healthy cousins. The family is of Turkish origin.

One of the proband’s half-sisters (II-1), a 16-year-old female whose chief complaints were delayed puberty and primary amenorrhea, was also diagnosed to have IHH. This patient was also given estrogen treatment elsewhere. Her height and weight were 158 cm (25^th^ percentile) and 60 kg (50-75^th^ percentile), respectively. Her sense of smell was normal. Her axillary and pubic hair were both at stage 2, while her breast Tanner stage was 3. Her karyotype was 46,XX. Her serum plasma estradiol, LH, and FSH levels were prepubertal (Table 1). Her parents were healthy and unrelated.

### Family 2

The proband (II-1) was an 18-year-old female patient who was also referred for lack of breast enlargement and primary amenorrhea. Her height and weight were 159 cm and 50.5 kg, respectively. Her axillary and pubic hair were at stage 3 while her breast Tanner stage was 2 bilaterally. Her sense of smell was normal. Her serum estradiol, FSH, and LH levels were 10.6 pg/mL, 1.0 mIU/mL, and 0.2 mIU/mL, respectively. Her karyotype was 46,XX. A GnRH stimulation test elicited peak levels of FSH and LH as 2.8 and 2.1 mIU/mL, respectively. Her brain MRI and pelvic ultrasonography results were normal.

One of her siblings (II-2), a 15-year-old boy, was also referred for delayed puberty. His testicular volumes were 2 mL bilaterally. Axillary and pubic hair were at stage 1. He reported a normal sense of smell. His reproductive hormone levels were prepubertal (Table 1). His karyotype was 46,XY. A GnRH stimulation test revealed peak LH and FSH levels of 10.4 and 7.5 mIU/mL, respectively. His cranial MRI was normal. The parents are healthy and of Turkish origin.

### Family 3

The proband (II-1) is a 14-year-old male patient who was referred for micropenis and absence of erections. He had a decreased sense of smell. Pubic and axillary hair were at stage 3. Testicular volumes were 2 mL bilaterally. He had no midline anomalies. His height and weight were 54 kg (25-50^th^ percentile) and 160 cm (25-50^th^ percentile), respectively. His basal testosterone and gonadotropin levels were prepubertal. His karyotype was 46,XY. His cranial MRI was normal. A paternal uncle (I-2) of his also reportedly suffers from absent puberty and anosmia. This family is ethnically Arabic.

## RESULTS

**Figure 1:**
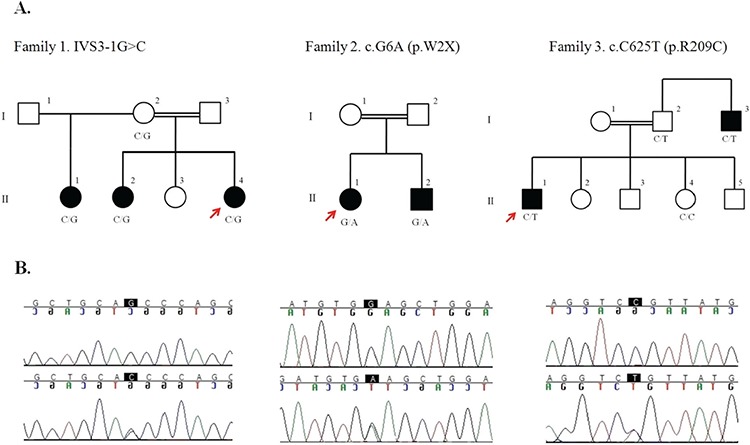
Segregation of the FGFR1 mutations in families with affected individuals. (A) Pedigrees and descriptions in each family are shown. Filled symbols show patients with normosmic idiopathic hypogonadotropic hypogonadism/Kallmann syndrome; open symbols show clinically unaffected individuals. Squares indicate male family members, circles indicate female family members, the double line indicates consanguinity, and arrows point to probands. Genotypes are shown below each tested family members. (B) DNA sequence analysis of patients. The positions of the mutations are marked. The top lines show the homozygous wild-type genotype, and the bottom lines show heterozygous genotype

A Sanger sequence analysis of the entire coding regions of *FGFR1* (HGNC:3688 NM_001174063, NP_001167534) revealed three novel mutations (Figure 1).

A whole exome sequencing on probands confirmed these variants but did not reveal any more potentially contributing variants in other known IHH/KS-associated genes.

The affected three sisters and their unaffected mother from family 1 were found to have the heterozygous IVS3-1G>C (g.G38886C) novel mutation. This splicing mutation in intron 3 is predicted to cause skipping of exon 4, eventually resulting in a totally different protein product as the last nucleotide of the exon 3 forms a codon with the first two nucleotides of exon 5. Stop codon formation occurred after 50 amino acids. Human Splicing Finder (www.umd.be), an *in silico* prediction program for splicing variants, predicted this variant as “alteration of the wild type acceptor site most probably affecting splicing”.

In family 2, affected siblings had the p.W2X (c.G6A) mutation in the heterozygous state. Parents were not available for testing.

In family 3, the heterozygous p.R209C (c.C625T) mutation was found in affected individuals as well as in the unaffected father of the proband. One hundred alleles from healthy individuals of Arabic origin did not show this variant.

Severe changes in protein structure and function were predicted in the splice site (IVS3-1G>C) mutation in family 1 and the nonsense mutation (p.W2X) in family 2. The mutation in family 3 (p.R209C) was predicted to be disease causing by Mutation Taster (http:/www.mutationtaster.org/) (probably damaging, PolyPhen-2 score:1.0) by PolyPhen-2 (http:/www.genetics.bwh.harvard.edu) and damaging by SIFT ( www.sift.jcvi.org). Conservation analysis showed that the arginine at 209 was highly conserved across species (Table 2).

## DISCUSSION

IHH is a term for heterogeneous disorders due to insufficient gonadotropin secretion. Most cases are referred with a delayed sexual maturation as teenagers. To date, more than 30 genes have been reported to be associated with IHH phenotype (3).

Herein, we report the results of screening for known genes associated with IHH/KS in a cohort of 52 patients. We identified three novel FGFR1 mutations including splicing, missense, and nonsense ones. The IVS3-1G>C (g.G38886C) mutation which involves the Ig-like C2-type 1 domain may cause total absence of the *FGFR1* gene product with premature stop codon that results in mRNA decay (12). To date, eight splicing-site mutations were reported in FGFR1. Six of the patients with splicing mutations (75%) were male (10,13,14). All of these patients were anosmic. Similar to our experience, Raivio et al (14) reported IHH patient with splicing mutation (c.C336T) which also affected exon 3. That anosmic patient was male and had a midline defect (corpus callosum agenesis). Our patients with splicing mutation were normosmic females and they had no symptoms other than those pertaining to the reproductive system. Gender differences may account for these remarkable phenotypic differences. Most notably, their heterozygous mother was reproductively healthy as evidenced by giving birth to four children without reproductive assistance. The normal reproductive phenotype in the mother despite having the same genotype as her affected daughters could be explained by reduced penetrance. This is also observed by others in families with IHH/KS due to FGFR1 mutations (15). These intra and inter-familial remarkable disassociations of phenotype and genotype necessitate further studies and probably indicate versatility of fibroblast growth factor signaling in GnRH ontogeny.

The proband in family 2 and her affected brother had a nonsense mutation (p.W2X, c.G6A) which causes a premature stop codon formation. This mutation is predicted to cause a total absence of protein product with its early occurrence. There are over a hundred different missense and nonsense mutations in the *FGFR1* gene. This novel mutation (p.W2X) affects the signal peptide which is in the initial point of this gene. Laitinen et al (16) reported three nonsense mutations (p.W4X, p.R609X, p.R262X) in the *FGFR1* gene. Their patient with p.W4X was a male who was diagnosed with KS without accompanying non-reproductive comorbidities. The p.W4X mutation is in close proximity to our mutation. Yet, both our patient and her affected brother had normosmic IHH. Different protective mechanisms or ethnic differences may account for the clinical inconsistency in terms of sense of smell.

Proband 3 and his affected uncle had a missense mutation p.R209C (c.C625T). This mutation affects Ig-like C2-type 2 domain which directly interacts with fibroblast growth factors and heparan sulfate proteoglycans (17). Only one patient with the exact nucleotide change resulting in p.R209C mutation has been previously reported by Tommiska et al (17). Their patient had micropenis and hyposmia. In our study, the affected individuals had a very similar phenotype. Laitinen et al (16) found a c.G626A mutation also resulting in the same aminoacid charge (i.e. p.R209C). This patient had also KS but no micropenis, suggesting a milder phenotype.

Concomitant whole exome sequencing data on probands did not reveal any more potentially contributing variants in other known IHH/KS-associated genes. In view of the oligogenic inheritance in IHH/KS (18), this finding is remarkable and may point to FGFR1 variants as the sole mediator of the phenotypes. However, the methods employed in this study cannot rule out copy number variations in the etiology as these variants have been shown to be important in KS (19). In conclusion, our results further substantiate great variability of reproductive and non-reproductive phenotype by various FGFR1 mutations. Expanding phenotype genotype catalogue in this pivotal gene may enhance our capability of clinical management as well as understanding *FGF* signaling.

## Figures and Tables

**Table 1:**
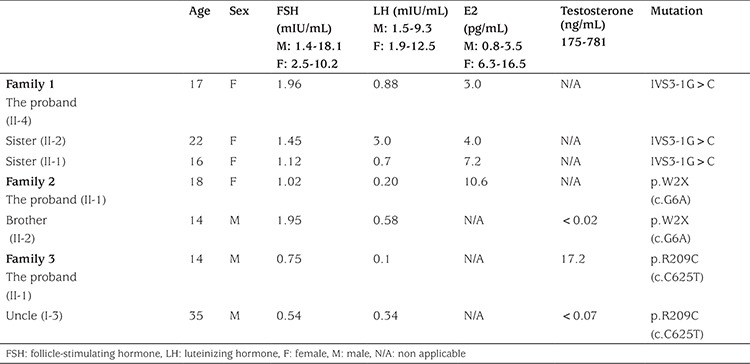
Clinical and laboratory characteristics of patients with FGFR1 mutations

**Table 2:**
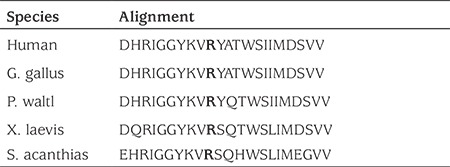
Evolutionary conservation of the mutated (p.R209C) FGFR1 amino acid across different species

## References

[1] Kaplan JD, Bernstein JA, Kwan A, Hudgins L (2010). Clues to an early diagnosis of Kallmann syndrome. Am J Med Genet A.

[2] Bianco SD, Kaiser UB (2009). The genetic and molecular basis of idiopathic hypogonadotropic hypogonadism. Nat Rev Endocrinol.

[3] Topaloglu AK, Kotan LD (2016). Genetics of Hypogonadotropic Hypogonadism. Endocr Dev.

[4] Schwanzel-Fukuda M, Bick D, Pfaff DW (1989). Luteinizing hormone-releasing hormone (LHRH)-expressing cells do not migrate normally in an inherited hypogonadal (Kallmann) syndrome. Brain Res Mol Brain Res.

[5] Dode C, Teixeira L, Levilliers J, Fouveaut C, Bouchard P, Kottler ML, Lespinasse J, Lienhardt-Roussie A, Mathieu M, Moerman A, Morgan G, Murat A, Toublanc JE, Wolczynski S, Delpech M, Petit C, Young J, Hardelin JP (2006). Kallmann syndrome: mutations in the genes encoding prokineticin-2 and prokineticin receptor-2. PLoS Genet.

[6] Hardelin JP, Dode C (2008). The complex genetics of Kallmann syndrome: KAL1, FGFR1, FGF8, PROKR2, PROK2, et al. Sex Dev.

[7] Gonzalez-Martinez D, Kim SH, Hu Y, Guimond S, Schofield J, Winyard P, Vannelli GB, Turnbull J, Bouloux PM (2004). Anosmin-1 modulates fibroblast growth factor receptor 1 signaling in human gonadotropin-releasing hormone olfactory neuroblasts through a heparan sulfate-dependent mechanism. J Neurosci.

[8] Ohkubo Y, Uchida AO, Shin D, Partanen J, Vaccarino FM (2004). Fibroblast growth factor receptor 1 is required for the proliferation of hippocampal progenitor cells and for hippocampal growth in mouse. J Neurosci.

[9] Gill JC, Tsai PS (2006). Expression of a dominant negative FGF receptor in developing GNRH1 neurons disrupts axon outgrowth and targeting to the median eminence. Biol Reprod.

[10] Dodé C, Fouveaut C, Mortier G, Janssens S, Bertherat J, Mahoudeau J, Kottler ML, Chabrolle C, Gancel A, François I, Devriendt K, Wolczynski S, Pugeat M, Pineiro-Garcia A, Murat A, Bouchard P, Young J, Delpech M, Hardelin JP (2007). Novel FGFR1 sequence variants in Kallmann syndrome, and genetic evidence that the FGFR1c isoform is required in olfactory bulb and palate morphogenesis. Hum Mutat.

[11] Choi M, Scholl UI, Ji W, Liu T, Tikhonova IR, Zumbo P, Nayir A, Bakkaloğlu A, Ozen S, Sanjad S, Nelson-Williams C, Farhi A, Mane S, Lifton RP (2009). Genetic diagnosis by whole exome capture and massively parallel DNA sequencing. Proc Natl Acad Sci U S A.

[12] Baker KE, Parker R (2004). Nonsense-mediated mRNA decay: terminating erroneous gene expression. Curr Opin Cell Biol.

[13] Laitinen EM, Tommiska J, Sane T, Vaaralahti K, Toppari J, Raivio T (2012). Reversible congenital hypogonadotropic hypogonadism in patients with CHD7, FGFR1 or GNRHR mutations. PLoS One.

[14] Raivio T, Avbelj M, McCabe MJ, Romero CJ, Dwyer AA, Tommiska J, Sykiotis GP, Gregory LC, Diaczok D, Tziaferi V, Elting MW, Padidela R, Plummer L, Martin C, Feng B, Zhang C, Zhou QY, Chen H, Mohammadi M, Quinton R, Sidis Y, Radovick S, Dattani MT, Pitteloud N (2012). Genetic overlap in Kallmann syndrome, combined pituitary hormone deficiency, and septo-optic dysplasia. J Clin Endocrinol Metab.

[15] Pitteloud N, Quinton R, Pearce S, Raivio T, Acierno J, Dwyer A, Plummer L, Hughes V, Seminara S, Cheng YZ, Li WP, Maccoll G, Eliseenkova AV, Olsen SK, Ibrahimi OA, Hayes FJ, Boepple P, Hall JE, Bouloux P, Mohammadi M, Crowley W (2007). Digenic mutations account for variable phenotypes in idiopathic hypogonadotropic hypogonadism. J Clin Invest.

[16] Laitinen EM, Vaaralahti K, Tommiska J, Eklund E, Tervaniemi M, Valanne L, Raivio T (2011). Incidence, phenotypic features and molecular genetics of Kallmann syndrome in Finland. Orphanet J Rare Dis.

[17] Tommiska J, Känsäkoski J, Christiansen P, Jørgensen N, Lawaetz JG, Juul A, Raivio T (2014). Genetics of congenital hypogonadotropic hypogonadism in Denmark. Eur J Med Genet.

[18] Sykiotis GP, Plummer L, Hughes VA, Au M, Durrani S, Nayak-Young S, Dwyer AA, Quinton R, Hall JE, Gusella JF, Seminara SB, Crowley WF, Pitteloud N (2010). Oligogenic basis of isolated gonadotropin-releasing hormone deficiency. Proc Natl Acad Sci U S A.

[19] Zhang SL, Tang YP, Wang T, Yang J, Rao K, Zhao LY, Zhu WZ, Meng XH, Wang SG, Liu JH, Yang WM, Ye ZQ (2011). Clinical assessment and genomic landscape of a consanguineous family with three Kallmann syndrome descendants. Asian J Androl.

